# CRISPR/Cas9-Based Antiviral Strategy: Current Status and the Potential Challenge

**DOI:** 10.3390/molecules24071349

**Published:** 2019-04-05

**Authors:** Choongho Lee

**Affiliations:** College of Pharmacy, Dongguk University, Goyang 10326, Korea; lkj640@gmail.com; Tel.: +82-31-961-5223

**Keywords:** CRISPR, Cas9, antiviral drug, efficacy, viral escape, resistance

## Abstract

From its unexpected discovery as a bacterial adaptive immune system to its countless applications as one of the most versatile gene-editing tools, the CRISPR/Cas9 system has revolutionized every field of life science. Virology is no exception to this ever-growing list of CRISPR/Cas9-based applications. Direct manipulation of a virus genome by CRISPR/Cas9 has enabled a systematic study of cis-elements and trans-elements encoded in a virus genome. In addition, this virus genome-specific mutagenesis by CRISPR/Cas9 was further funneled into the development of a novel class of antiviral therapy targeting many incurable chronic viral infections. In this review, a general concept on the CRISPR/Cas9-based antiviral strategy will be described first. To understand the current status of the CRISPR/Cas9-based antiviral approach, a series of recently published antiviral studies involving CRISPR/Cas9-mediated control of several clinically-relevant viruses including human immunodeficiency virus, hepatitis B virus, herpesviruses, human papillomavirus, and other viruses will be presented. Lastly, the potential challenge and future prospect for successful clinical translation of this CRISPR/Cas9-based antiviral method will be discussed.

## 1. Introduction

Along with a proteinaceous structural component, a nucleic acid is an essential building block for assembly of an infectious virus particle. Therefore, efficient viral genome replication inside a host cell is one of the most important tasks for the successful completion of a virus life cycle. In theory, the ablation of viral genetic elements has been regarded as one of the most ideal antiviral strategies. However, the lack of a virus gene-specific destruction method has been a big hurdle for the realization of this virus genome-targeting antiviral strategy. Recently, a variety of sequence-specific endonucleases have been introduced and tested for their therapeutic potentials for direct manipulation of a viral genome in preclinical studies. They include zinc finger nucleases, transcription activator-like effectors nucleases (TALENS), and clustered regulatory interspaced short palindromic repeat (CRISPR)-associated (Cas) nucleases [1,2]. Among them, the CRISPR/Cas system has been one of the most preferred choices for various antiviral applications due to its relative versatility, specificity, and ease of use [3,4,5,6,7,8]. Originally, CRISPR/Cas was discovered as one of the bacterial adaptive immune systems for defense against a foreign nucleic acid attack such as a phage infection and an exogenous plasmid uptake [9,10]. In order to achieve precise and specific digestion of these potentially harmful genetic elements from intruders, the CRISPR/Cas system was evolved to employ a foreign DNA-derived RNA as a guide molecule for sequence-specific destruction of target viral DNAs [11]. This RNA-directed sequence specificity of the CRISPR/Cas system has enabled a powerful and versatile genetic manipulation of genomes from diverse eukaryotic as well as prokaryotic organisms. Many successful applications to several human viral pathogens in cell-based and animal studies were highly encouraging, which is enough to hope for their accelerated translation in the clinical setting [3,4,5,6,7,8]. However, increasing concerns regarding the safety of CRISPR/Cas system due to its potential off-target activity and emergence of CRISPR/Cas-resistant escape mutant viruses along with the difficulty in its efficient delivery to every single virus-infected cell still seems to be a daunting task for full fruition of this promising antiviral approach [12,13]. In this review, the general concept regarding the design and efficacy validation method for CRISPR/Cas-based antiviral strategy will first be introduced and reviewed. Then, the current status of the CRISPR/Cas-based antiviral approach to control major pathogenic human viruses including human immunodeficiency virus (HIV), hepatitis B virus (HBV), herpes viruses, human papillomavirus (HPV), and other viruses will be summarized next. Lastly, this review will be concluded with thoughts regarding a potential challenge for the realization of CRISPR/Cas-based therapy and prospect for CRISPR/Cas-based antiviral strategy in the future.

## 2. CRISPR/Cas9-Mediated Antiviral Strategy

Type II CRISPR/Cas system, which is one of the most extensively characterized CRISPR/Cas systems, has a single effector DNA endonuclease called Cas9 [4]. Guidance of this endonuclease to its DNA target is mediated by two small RNAs including CRISPR RNA (crRNA) and trans-activating CRISPR RNA (tracrRNA) [11]. The invention of a single guide RNA (sgRNA), which is an artificially fused form of these two small RNAs, has made a great contribution to the evolution of CRISPR/Cas9 system as one of the most versatile and user-friendly gene-editing platforms ever developed [14,15]. A typical target site of the CRISPR/Cas9 system is composed of a 20-base pair-long seed sequence followed by three base pair-long proto-spacer adjacent motifs (PAM) (5′-NGG-3′). Upon satisfaction of the Watson and Crick sequence, complementarity between the CRISPR/Cas9 target site and the Cas9-bound sgRNA, Cas9-mediated double strand DNA break occurs, which is, in most cases, fixed by one of the host DNA repair systems and non-homologous end joining (NHEJ) reaction [16]. During this NHEJ-mediated DNA repair, random nucleotide insertion, deletion (Indel), and substitution around the DNA cleavage site take place, which leads to disruption of the essential virus protein-coding regions and/or cis-regulatory elements in a virus genome. This mutagenic effect of NHEJ-mediated DNA repair on a virus genome results in blockage of virus genome replication, which leads to the ultimate establishment of the antiviral status inside a host cell.

**Figure 1:**
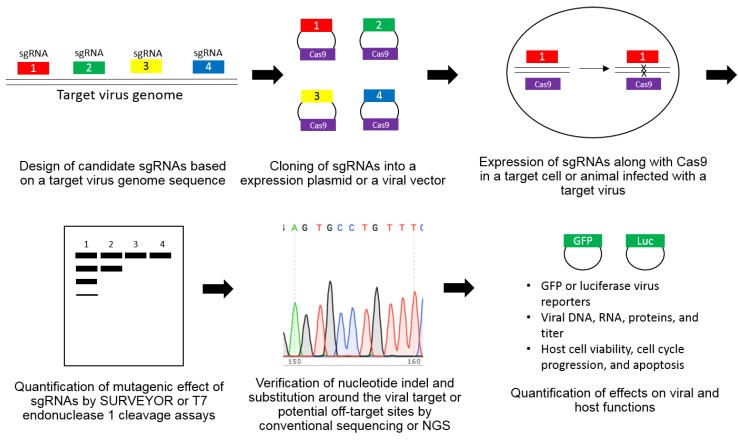
A step-by-step description of a typical CRISPR/Cas9-based antiviral study.

In order to test the antiviral efficacy of the CRISPR/Cas9 system in any type of viral disease, candidate sgRNAs need to be designed and constructed based on a target virus genome sequence (Figure 1). Then, these virus genome-targeting sgRNAs need to be cloned into an expression plasmid or a viral vector. Together with Cas9 protein, they are expressed via either a plasmid-based transfection or lentivirus/adenovirus-based transduction methods. The cleavage-induced mutations around the viral target sequence are detected and quantitated by either SURVEYOR or T7 endonuclease I cleavage assays by using the ability of T7 endonuclease I to recognize and cleave non-perfectly matched DNAs. Conventional Sanger or next-generation sequencing (NGS) confirms the presence of cleavage-induced mutations in a virus genome. The off-target activity of the CRISPR/Cas9 system is also studied by examining the potential host DNA target sequences with relatively high sequence homology to a viral target sequence. In general, their antiviral efficacy is assessed by using either a GFP or luciferase-based reporter virus or a direct quantification of viral DNA, RNA, and protein levels. Effects on host cells such as cell viability, apoptosis, and cell cycle progression are also studied to detect any undesirable changes induced by CRISPR/Cas9-mediated destruction of a target virus genome. The long-term effect of CRISPR/Cas9-based alteration on a virus genome is also analyzed for detecting any CRISPR/Cas9-resistant escape mutant viruses.

## 3. Current Status of CRISPR/Cas9-Mediated Antiviral Strategy

Most of the current antiviral therapies for control of chronic viral infections by HIV, HPV, herpesviruses, and HPV failed to achieve a clinical cure due to their inherent inability to clear a virus genome from an infected host cell due to a latency state during which these viruses minimize its activity inside a host cell to avoid a host immune surveillance. The latency-related life cycle of these viruses plays a critical role in incurability of chronic infections induced by these viruses. Therefore, patients infected with these viruses need to take a life-long antiviral medication. In this regard, CRISPR/Cas9 technology holds great promise as a curative therapy for chronic infection. In order to understand the current status of this CRISPR/Cas9-based antiviral strategy, a series of recently published studies on CRISPR/Cas9-mediated control of HIV, HBV, herpesvirus, HPV, and other viruses will be presented as follows.

### 3.1. HIV

Highly active antiretroviral therapy (HAART) transformed an acquired immunodeficiency syndrome (AIDS) from a form of the deadly and hard-to-treat disease to a treatable and manageable medical condition. However, its life-long duration and ultimate incurability have been a great burden on many AIDS patients [1]. Therefore, there has been an urgent need for the development of a new antiviral strategy for a permanent cure for HIV infection. This incurability of HIV infection by HAART is mainly due to its inability to remove the chromosomally-integrated viral DNAs. In this regard, CRISPR/Cas9 technology seems to be best suited for the knockout task of these inserted viral genetic elements to achieve a “sterile” cure of HIV infection. Many studies demonstrated successful ablation of an HIV genome by using the CRISPR/Cas9 technique both in vitro and in vivo settings [2,3,4,5,6,7,8,9,10,11,12]. In addition, this CRISPR/Cas9-based gene-editing was further applied to a disruption of essential HIV host dependency factors such as HIV co-receptors, chemokine receptor type 5 (CCR5), and C-X-C chemokine receptor type 4 (CXCR4) [13,14,15,16,17,18,19,20,21,22,23,24,25]. In general, a host-targeting antiviral approach is thought to be less likely to develop viral resistance. Therefore, suppression of host dependency factors by the CRISPR/Cas9 system is expected to circumvent the problem of generating CRISPR/Cas9-resistant viral mutants.

Chromosomal integration-initiated HIV latency, subsequent minimal transcription of the HIV genome, and consequent preservation of HIV reservoirs are well-characterized hallmarks of HIV infection [26]. In order to disrupt this HIV latency state, the so-called “shock and kill” anti-HIV strategy was proposed. This antiviral strategy involves induction of latency reversal through either genetically-reinforced or pharmacologically-reinforced viral promoter activation [27]. For the application of CRISPR/Cas9 to this “shock and kill” anti-HIV approach, the sequence-specific targeting ability of Cas9 was further harnessed to develop a catalytically-dead version of Cas9 (Cas9d). This special version of Cas9 has been equipped with the capability of a virus promoter-specific transcriptional activation through its artificial association with exogenous transcriptional activation domains [28,29,30]. This CRISPR/Cas9d-based viral promoter-activating antiviral approach showed promising antiviral efficacy [28,29,30,31,32]. On the other hand, induction of host restriction factors such as interferons and interferon-stimulated genes (ISG) by CRISPR/Cas9-mediated transcriptional activation was envisaged as another plausible approach for this host-targeting antiviral strategy [33,34]. In addition, a number of anti-HIV applications of CRISPR/Cas9 involving the enhanced expression of host restriction factors against HIV infection such as apolipoprotein B mRNA editing enzyme, catalytic polypeptide-like (APOBEC3G), and tripartite motif-containing protein 5 alpha (TRIM5α) genes through Cas9d-mediated transcriptional activation were also proposed and tested [33,34]. In the following chapter, different kinds of CRISPR/Cas9-based anti-HIV strategies such as direct disruption of an HIV genome, induction of latency reversal, disruption of a host dependency factor, and induction of a host restriction factor will be discussed in detail. In addition, the development of viral escape mutants to CRISPR/Cas9-based antiviral therapy and how to control these viral escape mutants will be described later.

#### 3.1.1. Direct Disruption of an HIV Genome

Eleven papers reported successful anti-HIV applications of CRISPR/Cas9 system through direct disruption of an HIV genome [2,3,4,5,6,7,8,9,10,11,12] (Table 1). Although most of the studies used Cas9 from *Streptococcus pyogenes*, which is the most widely utilized one [3,4,6,8,12], five studies employed Cas9 from *Staphylococcus aureus*, which has a better viral particle production efficiency due to its smaller size [2,5,9,10,11]. For CRISPR/Cas9 delivery, most of the studies used a lipofectamine-based transfection while some used lentiviral [2,6,7,9,10,11] or adenoviral transductions [5,11]. Different regions of the HIV-1 genome such as a long terminal repeat (LTR) and other viral protein-coding sites (*gag*, *pol*, *env*, and other accessory genes) were chosen for the synthesis of a panel of gRNAs with an LTR region being the most preferred target site. For CRISPR/Cas9-mediated cleavage efficacy screening, various forms of LTR-driven GFP or luciferase reporter systems were employed. Upon introduction of the CRISPR/Cas9, most of the studies reported efficient cleavage of target sites and mutagenic effect on a virus genome, which turned out to be translated into a potent antiviral efficacy [2,3,4,5,6,7,8,9,10,11,12]. Their antiviral potentials were further demonstrated by decreased GFP [3,4,6,12] or luciferase expression of a virus reporter gene [7,10,11], reduced latency reactivation [3,4,6,7], decreased viral copy number [2,4,5,6,7,11], diminished production of a viral protein, p24 [2,4,6,8,9,12], and immunization to a new HIV infection [4,6,7,8,9]. In addition, most of the studies reported no off-target cleavage around top-ranked CRISPR/Cas9 recognition sites inside a host genome [3,4,6,8,9,11]. Some studies reported no significant effect on cell viability [4,6]. In particular, two studies reported the in vivo antiviral activity of the CRISPR/Cas9 system against HIV-1 genome replication by using HIV-1 Tg26 transgenic and humanized bone marrow/liver/thymus mice [5,11]. Based on these results, CRISPR-Cas9-mediated disruption of an HIV genome was concluded as a very effective antiviral strategy.

#### 3.1.2. Induction of Latency Reversal (Shock and Kill Strategy)

Five papers reported successful anti-HIV applications of the CRISPR/Cas9 system by inducing latency reversal (Table 1) [28,29,30,31,32]. For selective, potent, and persistent reactivation of the HIV-1 latent reservoirs through transcriptional activation of a viral LTR promoter, two different kinds of CRISPR/Cas9d systems were employed based on transcriptional activation domains such as MS2-p65-HSF1-synergistic activation mediator (SAM) and VP64-SunTag [28,29,30,31,32]. For delivery of CRISPR/Cas9d system, most of the studies used a lipofectamine-based transfection [28,29,31,32] while some used lentiviral transduction [29,30]. For screening of CRISPR/Cas9-mediated LTR activation, different versions of LTR-driven GFP or luciferase reporter systems were employed. Efficient transcriptional activation of a viral promoter by the CRISPR/Cas9 system was manifested by an increased GFP [28,29,30,31], luciferase expression of a viral promoter reporter [28,30,31,32], and enhanced production of the p24 viral protein [28,31]. Elevated production of infectious particles [28] and antiviral synergy with other latency reversal agents such as suberoylanilide hydroxamic acid (SAHA) and prostratin [29] was also observed. As evidence for the expected antiviral mode of action, suicidal cell death due to a buildup of toxic viral proteins was also noticed [30]. Of note, no adverse effect or genotoxicity was reported by this approach [31,32]. Based on these results, a latency reversal via CRISPR/Cas9-assisted intentional activation of an HIV promoter seems to be another effective CRISPR/Cas9-mediated antiviral strategy against HIV infection.

#### 3.1.3. Disruption of a Host Dependency Factor

Aside from a direct attack on a viral genome, the CRISPR/Cas9 system can be applied to the abrogation of host dependency factors, which are required for any critical steps in the virus’ life cycle. As previously mentioned, this host-targeting antiviral strategy has been shown to be less likely to generate drug-resistant mutant viruses during the course of antiviral treatment over conventional virus-targeting antivirals. Suppression of an HIV entry by disruption of its co-receptors, CCR5 and CXCR4 is one of the most typical examples for this host-targeting antiviral approach [18,19,20]. So far, fourteen studies described inhibition of HIV infection by disrupting essential host dependency factors required for an HIV infection (Table 1) [13,14,15,16,17,18,19,20,21,22,23,24,25,35]. These CRISPR/Cas9-targeted HIV host dependency factors include HIV co-receptors, CCR5 [13,14,15,17,18,20,22,23,24,25] and CXCR4 [16,17,19,20,21,25], and microRNA-146 [35]. For CRISPR/Cas9 delivery, most studies used a lipofectamine-based transfection [13,14,15,24,25,35] while some used a lentiviral [16,18,20,21,22,25,35], adenoviral transduction [18,21], and even electroporation [17,20,25]. In particular, three studies reported a CRISPR/Cas9-mediated simultaneous disruption of both CCR5 and CXCR4 [17,20,25]. Co-receptor tropism-specific resistance to HIV-1 infection was demonstrated in most of the single CCR5 or CXCR4 disruption studies [18,19,20,21,22,24,25]. In particular, one study demonstrated the in vivo antiviral activity of the CRISPR/Cas9 system targeting CCR5 by using NOD/Prkdc-scid/IL-2Rγnull mice [23]. Expression of a GFP reporter virus [16,17,21] or p24 viral protein [16,18,19,20,21,25], and viral RNA load [16,19,23,35] were all reduced by ablation of HIV co-receptors by CRISPR/Cas9. The elimination of microRNA-146α by CRISPR/Cas9 led to a marked increase in the expression levels of cytokines and HIV-1 restriction factors [35]. Although one study pointed out off-target activity in a high-homology host gene such as CCR2 [15], most of the studies reported no significant off-target activity [14,16,18,19,20,21,22,24,25,35]. Selected survival advantage of co-receptor-disrupted cells was also noticed [20,22]. No apoptosis, genotoxicity, or cytotoxicity was reported in most of the studies [16,19,20,21,25]. All these results indicate the antiviral potential for CRISPR/Cas9-based disruption of key host factors required for HIV infection.

#### 3.1.4. Induction of a Host Restriction Factor

Two studies described ablation of the HIV genome through specific CRISPR/Cas9-assisted activation of host restriction factors such as APOBEC3 and TRIM5α for HIV infection (Table 1) [33,34]. APOBEC3 was shown to work as an anti-HIV host factor through its virus-specific mutagenic activity against an HIV genome [43,44]. The anti-retroviral activity of TRIM5α was also shown to be mediated by its destabilization and sequestration of the viral capsid core proteins [45,46]. In particular, the introduction of two amino acid substitutions known as R332G and R355G in the human TRIM5α domain converted it to a genuine restriction factor for HIV-1 infection [47]. In the study conducted by Boger et al., they utilized a single sgRNA, which was modified to contain MS2-derived stem-loops. These MS2-derived stem-loops were able to recruit fusion proteins consisting of the MS2 coat protein linked to transcription activation domains, which results in the induction of an otherwise silent cellular APOBEC3 gene [33]. Consequently, upregulated expression of APOBEC3 gene by CRISPR/Cas9 led to decreased luciferase expression of an HIV reporter gene and reduced viral infectivity due to an APOBEC3-induced mutation in the HIV genome [33]. In case of the TRIM5α-targeting approach by CRISPR/Cas9, Dufour et al. tried to use CRISPR/Cas9 for the mutation of TRIM5α to its potentially HIV-1-restrictive version by a homology-directed repair (HDR) [34]. Unfortunately, no significant antiviral effects were observed in TRIM5αR332G-targeted cells due to the presence of undesired additional mutations [34].

#### 3.1.5. Generation of the Viral Escape Mutant and the Development of Resistance

In spite of potent inhibition of HIV-1 replication by CRISPR/Cas9 system, HIV-1 has been shown to be able to generate escape mutants from a single antiviral gRNA by NHEJ-mediated modification of the target sequence (Table 1) [36,37,38,39,40,41,42]. Development of viral resistance to this monoplex gRNA approach seems to bear a similarity to the development of drug resistance to single antiretroviral therapy. Heterogeneity in the populations of an HIV genome has been linked to an error-prone nature of a viral reverse transcriptase. In the clinical setting, most anti-HIV HAART therapy is based on a combined regimen composed of more than two different classes of anti-HIV drugs with a different mechanism of actions for effective control of HIV quasi-species. In line with this concept, a multiplex approach, which targets several different regions of an HIV genome in a simultaneous fashion, turned out to be more effective for suppressing the development of viral resistance to CRISPR/Cas9 than the monoplex one [38,41]. In the study performed by Lebbink et al., they demonstrated complete abrogation of viral replication and prevention of a viral escape by a combinatorial approach of two strong gRNAs targeting different regions of an HIV genome [41]. Wang et al. also found a delayed viral escape through combinations of two separate gRNAs, and identified two gRNA combinations with the highest competency for durable blockage of HIV-1 replication [38]. Based on these findings, the CRISPR/Cas9-based antiviral strategy was suggested to be executed in a combinatorial manner in order to prevent the development of CRISPR/Cas9-resistant viral escape mutants.

### 3.2. HBV

More than 240 million people around the world still suffer from chronic HBV infection [48,49]. The current HBV treatment regime mainly relies on the use of nucleoside and nucleotide analogs, which are reverse transcriptase inhibitors [50]. Similar to HIV patients, these anti-HBV therapeutics are not able to provide a cure for HBV infection. This incurable nature of the current anti-HBV treatment is due to their inability for removal of the stable nuclear covalently closed circular DNA (cccDNA), which serves as a transcription template for viral mRNA and pre-genomic RNA synthesis [51]. Therefore, manipulation of HBV cccDNA by the CRISPR/Cas9 system seems to be a perfect therapeutic application for this gene-editing technology [52,53,54,55,56,57,58,59,60].

Nineteen papers reported successful anti-HBV applications of the CRISPR/Cas9 system through direct disruption of the HBV genome (Table 2) [61,62,63,64,65,66,67,68,69,70,71,72,73,74,75,76,77,78,79]. Most of the studies used Cas9 from *Streptococcus pyogenes* [61,62,63,65,66,67,68,69,71,72,73,74,76,78], while two studies employed a smaller version of Cas9 from *Staphylococcus aureus* (saCas9) [75,77]. Three studies used Cas9nickase [64,70,79], which has a reduced off-target activity due to its induction of a single strand DNA break instead of a double-stranded one [80,81]. For CRISPR/Cas9 delivery, most of the studies used a lipofectamine-based transfection [61,63,64,66,67,68,69,70,72,74,76,79] while some used a lentiviral [62,65,66,67,69,71] or adenoviral transduction [75,77,78]. In particular, this includes the use of the adeno-associated virus (AAV) as a delivery vehicle for a smaller version of Cas9, saCas9 by two studies [75,77]. They demonstrated a similar anti-HBV efficacy with an enhanced capacity for production of a high-titer virus, which enables a potential delivery of CRISPR/Cas9 components into every single HBV-infected cell in the patient [75,77]. In the case of in vivo experiments, the hydrodynamic injection (HDI) was the most frequently used for delivery of CRISPR/Cas9 as well as HBV DNAs [61,63,66,67,69,76,77]. One study used a lipid-like nanoparticle as a delivery vehicle [73]. Different regions of the HBV genome encoding surface antigen, X protein, core, and polymerase were chosen for the synthesis of a panel of gRNAs. For CRISPR/Cas9-mediated cleavage efficacy screening, HBV DNA and mRNA together with viral proteins such as hepatitis B core antigen (HBcAg), hepatitis B surface antigen (HBsAg), and hepatitis B e antigen (HBeAg) were quantified. Upon introduction of the CRISPR/Cas9 system targeting different regions of an HBV genome, all studies reported a decreased level of HBcAg and HBsAg viral proteins [61,62,63,64,65,66,67,68,69,70,71,72,73,74,75,76,77,78,79]. Furthermore, gRNAs targeting the conserved HBV sequence turned out to be more effective for the suppression of HBV genomes of different genotypes than those targeting the less conserved region [61]. Karimova et al. showed CRISPR/Cas9-mediated disruption of not only episomal cccDNA but also chromosomally integrated HBV sequences in reporter cell lines [64]. Reduction of cccDNA was also reported by most of the studies [63,65,67,68,69,74,75,76,77,78]. In vivo antiviral efficacy by CRISPR/Cas9 was also demonstrated in several studies using an HDI mouse model [61,63,66,67,69,76,77]. Enhanced inhibition of HBV DNA accumulation by a currently used anti-HBV drug in combination with Cas9/sgRNAs suggests a potential combination of a pharmacological and gene-targeting approach for the induction of maximal antiviral potency [65]. Wang et al. even tried simultaneous expression of two gRNAs and miR-HBV by using a gRNA-miR-HBV-gRNA ternary cassette and confirmed their strong inhibition of HBV replication [76]. In addition, fives studies reported no off-target cleavage around top-ranked potential CRISPR/Cas9 recognition sites in the host genome [70,74,75,77,78]. Some studies reported no significant effect on cell viability [66,68]. Several multiplex approaches involving the excision of an HBV genome was also demonstrated to confer increased antiviral efficacy on the HBV genome [68,70,76,78]. Disruption of HBsAg by CRISPR/Cas9 led to the inhibition of proliferation and tumorigenicity of HBV-positive hepatocellular carcinoma cells, which further suggests the HBV-targeting CRISPR/Cas9 approach as an anti-cancer agent against an HBV-induced liver cancer [79]. Based on these results, CRISPR-Cas9-mediated disruption of the HBV genome seems to be a very effective virus-targeting antiviral strategy with the potential for combination with a current anti-HBV regimen.

### 3.3. Herpes Viruses

More than 90% of the adult population suffers from one or multiple forms of herpes virus infection [82]. The most well-known characteristics of the herpes virus infection is a chronic establishment of latency due to the inability of a host cell to clear the invader from infected cells, which ultimately results in a lifelong infection [83]. Herpes viruses including the herpes simplex virus type (HSV) 1, the Epstein Barr virus (EBV), and the human cytomegalovirus (HCMV) are responsible for a wide variety of recurrent diseases such as cold sores, shingles, congenital defects, and several malignancies [84]. Although the productive phase of a herpes virus infection can often be efficiently controlled by DNA polymerase inhibitors, these drugs are not able to remove herpes viruses from the human host during a latent phase of the herpes virus infection [85]. Therefore, in order to achieve a functional cure for the herpes virus infection, direct ablation of a herpes virus genome is thought to be the most ideal antiviral approach.

#### 3.3.1. HSV-1

HSV-1 is a human neurotropic virus responsible for significant morbidity and mortality with no permanent curative therapy [86,87]. Two papers reported successful applications of CRISPR/Cas9 for removal of an HSV-1 genome [88,89] (Table 3). Roehm et al. showed efficient inhibition of HSV-1 replication by CRISPR/Cas9-based targeting of ICP0, which is a key viral protein necessary for stimulation of HSV-1 gene expression [89]. In this paper, they observed the reversal of HSV-1-induced disintegration of promonocytic leukemia nuclear bodies by CRISPR/Cas9-mediated disruption of ICP0 [89]. Xu et al. targeted another viral protein called UL7, which is a tegument protein of HSV-1. They found diminished genome replication, attenuated neuro-virulence, and decreased pathologic effect by HSV-1 [88]. In the latency model, the expression of the latency-associated viral transcript was also lowered by CRISPR/Cas9-mediated targeting of UL7 [88]. In particular, CRISPR/Cas9-mediated knock-out of a UL7 gene resulted in reduced transcription of the immediate-early gene α-4 [88]. Based on these results, they demonstrated the necessity of the viral gene UL7 for efficient HSV-1 replication and virulence.

#### 3.3.2. EBV

EBV infection is responsible for the development of mononucleosis and is associated with certain types of lymphoma including Burkitt’s lymphoma, Hodgkin’s disease, nasopharyngeal carcinoma, and gastric cancer [90]. To date, no effective EBV vaccine or treatment is available.

Three papers described CRISPR/Cas9-based disruption of the EBV genome (Table 3) [91,92,93]. Want et al. found dramatic proliferation arrest and the concomitant reduction in viral loads in patient-derived cells from a Burkitt’s lymphoma with latent EBV infection after CRISPR/Cas9-mediated targeting of EBV nuclear antigen (EBNA) and latent membrane protein (LMP) regions of an EBV genome [91]. Yuen et al. used two gRNAs for deletion of 558 bp in the promoter region of BART (BamHI A rightward transcript), which encodes viral microRNAs [92]. In this study, they found a decreased expression of miR-Bart3 and declined viral yields in latently-infected EBV models. The same group also reported down-regulation of EBV DNA loads and lytic replication in latently-infected nasopharyngeal carcinoma cells by using the CRISPR/Cas9 technology [93]. The suppression of EBV DNA load further sensitized EBV-positive carcinoma cells to chemotherapeutic killing by anti-cancer agents such as cisplatin and 5-fluorouracil [93], which raises the possibility of combined use of the CRISPR/Cas9-based antiviral approach with a conventional anti-neoplastic agent.

#### 3.3.3. Human Cytomegalovirus (HCMV)

HCMV is an etiological agent for causing severe pathologies in non-immunocompetent patients [90,94]. In particular, HCMV infection is the major source of transplant-related morbidity and mortality. One study reported impairment of HCMV replication by excision of an essential HCMV gene, UL122/123, through the multiplex CRISPR/Cas9 approach (Table 3) [95]. In this paper, they found more efficient disruption of multiple viral functions including viral immediate-early protein expression, genome replication, late protein expression, particle production, and envelope glycoprotein B protein expression by simultaneous multiple targeting of different regions of a virus genome, which further emphasizes the superiority of the multi-targeting strategy over a single targeting strategy [95].

#### 3.3.4. Viral Escape and Resistance

Van Diemen et al. applied CRISPR/Cas9 for negative manipulation of three herpes viruses including HSV-1, EBV, and HCMV (Table 3) [96]. In this paper, they demonstrated effective abrogation of HCMV and HSV-1 replication by targeting gRNAs to essential viral genes such as BARTs and EBNA 1. Simultaneous targeting of HSV-1 with multiple gRNAs, once again, resulted in complete abolition of the production of infectious viral particles [96]. In addition, complete clearance of EBV from latently infected human tumor cells was also achieved [96]. In particular, inefficient targeting of HCMV by single gRNAs led to the selection for viral escape mutants after prolonged replication [96]. These data further highlight the importance of multiplex targeting of different viral genome regions for the prevention of escape mutant viruses resistant to CRISPR/Cas9 digestion.

### 3.4. HPV

High-risk type HPV infection is responsible for the development of cervical cancers in women. In spite of the successful development of different kinds of prophylactic vaccines against HPV infection, HPV-specific antiviral drug is not yet available. Seven papers reported successful anti-HPV applications of CRISPR/Cas9 system through direct disruption of the HPV genome (Table 4) [97,98,99,100,101,102,103]. Although most of the studies used Cas9 from *Streptococcus pyogenes*, one study employed a smaller version of Cas9 from *Staphylococcus aureus* [103]. For CRISPR/Cas9 delivery, most of the studies used a lipofectamine-based transfection [97,99,100,101,102] while two used a lentiviral [98] or adenoviral transduction [103]. Two different regions of an HPV genome such as E6 and E7 were chosen for the synthesis of a panel of gRNAs due to their biological importance as viral oncogenes. For CRISPR/Cas9-mediated cleavage efficacy screening, HPV DNA and mRNA together with viral proteins such as E6 and E7 were quantified. Upon introduction of the CRISPR/Cas9 system, some studies reported a decreased level of E6 or E7 mRNA or protein [97,99,100]. Inactivation of E6 and E7 genes were further demonstrated by the recovery of tumor suppressor genes such as p53, retinoblastoma protein (pRb), and p21 [97,98,99,101,102]. Consequently, cell or tumor growth inhibition was also demonstrated in all studies targeting HPV by CRISPR/Cas9 [97,98,99,100,101,102,103]. In addition, one study reported no off-target cleavage around top-ranked potential CRISPR/Cas9 recognition sites [98]. Based on these results, CRISPR-Cas9-mediated disruption of an HPV genome can be regarded as one of the most effective antiviral approaches against HPV infection.

### 3.5. Polyoma JC Virus

The human neurotropic polyomavirus, JC (JCV), is responsible for the development of the fatal demyelinating disease progressive multifocal leukoencephalopathy (PML) [104]. As of now, there is no cure for PML and, in most cases, disease progression leads to death within two years. Wollebo et al. showed abrogation of the expression of the viral proteins such as T-antigen, VP1, and agno-protein and subsequent suppression of viral replication in permissive cells by the expression of Cas9 and gRNAs specifically targeting the viral T-antigen (Table 5) [105]. This raises hope for the potential use of CRISPR/Cas9 as a new anti-JCV therapeutic in the near future.

### 3.6. African Swine Fever Virus

African swine fever (ASF) is an economically important infectious disease of swine with high mortality rates. This disease is caused by an African swine fever virus (ASFV) with the double-stranded DNA genome [106]. Hubner et al. observed complete abrogation of ASFV yields by targeting the viral phosphoprotein p30 (Table 5) [107]. However, during their continuous passage experiments, they found resistant ASFV mutants with one or two nucleotide exchanges leading to one or two amino acid substitutions [107]. Once again, this result reiterates the necessity of the multi-targeting CRISPR/Cas9 approach for complete suppression of escape variant mutant virus generation.

### 3.7. Pseudorabies Virus

Pseudorabies virus (PRV), which is a swine herpes virus, causes significant morbidity and mortality in swine populations, which results in huge economic losses in the global swine industry [108]. Peng et al. used sgRNAs targeting the conserved UL30 gene of PRV for the disruption of PRV replication (Table 5) [109]. However, they found significant restoration of early decline in viral copy number and titer to a normal level in a passage-dependent manner [109]. In addition, they showed resistance to CRISPR/Cas9-mediated digestion through nucleotide substitutions around the UL30 gene target site after cleavage [109]. In the follow-up study conducted by Tang et al., the author also confirmed the generation of escape mutants by single sgRNAs and the achievement of complete inhibition of PRV replication by multiple sgRNAs, which further highlights the importance of multiple targeting [110].

### 3.8. Hepatitis C Virus

Hepatitis C is an inflammatory liver disease caused by infection of hepatocytes with a hepatotropic single-stranded RNA virus known as the hepatitis C virus (HCV). Around 170 million people are estimated to be infected with HCV worldwide [111]. Thanks to dramatic progress in the development of many effective direct antiviral agents (DAA) targeting viral proteins, most HCV infections from different genotypes are now curable with appropriate pharmacological intervention. This might put some doubts about the role of CRISPR/Cas9-based therapy against HCV in the future. However, there is still a chance for development of drug-resistant mutant HCV variants, which may not be manageable by the current anti-HCV regimen. In this scenario, the CRISPR/Cas9-mediated disruption of the HCV genome might serve as an alternative antiviral strategy. In this perspective, a relevant previous work revealed a unique ability of Cas9 from *Francisella novicida* (FnCas9) to target a bacterial mRNA, which leads to the repression of a viral gene [112]. By taking advantage of this RNA-targeting capability of FnCas9, Price et al. showed the inhibitory activity of the CRISPR/FnCas9 system against an HCV RNA genome within eukaryotic cells (Table 5) [113]. They found an inhibition of HCV translation by a catalytically inactive version of FnCas9 (D11A/H969A). This suggests no need for direct digestion of viral RNAs for the complete shutdown of viral protein translation by the CRISPR/FnCas9 system [113]. Instead, the association of FnCas9 with an HCV RNA genome turned out to be sufficient for the suppression of both viral translation and genome replication [113]. Based on these results, the requirement for the application of CRISPR/Cas9 to an RNA virus seems to be very different from that of a DNA virus.

## 4. Potential Challenges for CRISPR/Cas-Based Antiviral Therapy

So far, numerous successful applications of CRISPR/Cas9 technology to anti-viral manipulation of major viral diseases were presented. However, there seem to be many hurdles to overcome for its realization in human application. First, its potential off-target activity needs to be minimized to ensure safety in an in vivo application. Erroneous digestion of an essential host gene with a high degree of homology to the 20 bp seeding plus PAM sequence of the CRISPR/Cas9 target site could result in a catastrophic outcome in a host cell. For a tight control of this potential off-target activity of CRISPR/Cas9 system, a different version of Cas9, called Cas9nickase, which has a reduced off-target property due to its induction of a single strand DNA cleavage instead of a double strand one, is highly recommended for human use [80,81]. Recently, three CRISPR/Cas systems with high gene-editing efficiencies with low off-target cleavage should be a much safer choice for clinical application of a CRISPR/Cas9-based antiviral strategy [114,115,116]. Second, delivery of CRISPR/Cas9 components into every virus-infected cell is another big challenge that needs to be resolved for successful clinical translation of this new technology since the presence of uncorrected residual virus-infected cells could serve as another viral reservoir for spreading a new virion to already-corrected normal cells. In this regard, AAV emerges as an ideal delivery tool thanks to its high viral titer capability with potential for full transduction of all virus-infected cells within a patient [117]. Targeted delivery of AAV to a certain tissue could be achieved by recombinant engineering of an AAV capsid protein with a tissue tropism for an intended infection site. In addition, an established record of safety and no integration property of AAV seems to be an ideal delivery method for CRISPR/Cas9 [117]. In line with this, several groups already demonstrate the feasibility of this AAV delivery method in CRISPR/Cas9-based antiviral studies [5,11,21,75,77,103]. In spite of their wide use in the in vitro and in vivo delivery of the CRISPR/Cas9 system, viral vectors possess several inherent problems including integration-induced disruption of host genes and subsequent development of cancer, size restriction in foreign gene insertion, potential immune responses, and difficulty in mass production. In order to circumvent these viral vectors-associated limitations, the development of non-viral vectors such as lipid-based or polymer-based nano-carriers has shown great promise as an alternative delivery method for CRISPR/Cas9 [26,73,118]. Third, the development of viral resistance to the CRISPR/Cas9 system through the generation of a viral escape mutant is another concern needed to be addressed before its translation into clinical practice. In order to avoid this phenomenon, careful selection of the most conserved and essential regions of a virus genome for the design of gRNA is absolutely required. The efficient CRISPR/Cas9-mediated control of a viral infection caused by a population of viruses with a diverse sequence variation (quasi-species) requires the most rigorous and optimal selection of gRNAs. In addition, many single gRNA-based monoplex approaches were shown to lose its antiviral efficacy due to the selection of a mutant virus variant with a modified target site, which is no longer cleavable by the CRISPR/Cas9 system [36,37,38,39,40,41,42,96,107,110]. Based on these observations, the multiplex approach turned out to be very effective for the suppression of the generation of a virus escape mutant and long-term maintenance of antiviral activity. Fourth, the safety of CRISPR/Cas9-based host-targeting antiviral approach needs to be validated in a more rigorous fashion. Abolishment of host factors required for a certain step of a virus life cycle was suggested as one typical example of indirect applications of the CRISPR/Cas9 system. This CRISPR/Cas9-assisted host-targeting seems to enjoy the benefit of the much reduced viral resistance relative to direct modification of a virus genome. However, the selection of a host dependency factor, which is both indispensable for a virus and dispensable for a host, is not an easy task. In addition, a detailed analysis of the host immune response to the CRISPR/Cas system needs to be conducted for the prediction of potential side effects associated with this antiviral therapy. In a practical point of view, the price of this CRISPR/Cas9-based antiviral therapy needs to be in a reasonably affordable range for the benefit of most infected patients.

## 5. Conclusions

Tight linkage of a viral life cycle to a host cellular metabolism has made clearance of this invader out of our body a therapeutic challenge. Pharmacological intervention of viral infection by using a small molecule inhibitor against a virus-specific enzyme has been one of the best antiviral options so far. However, this conventional antiviral approach has been inappropriate for the control of most of the latency-associated chronic viral infections. In this regard, the introduction of the CRISPR/Cas technology with an unprecedented capability for direct targeting of a viral genome would contribute to the buildup of a new antiviral armamentarium aiming for a previously unthinkable, complete cure. In spite of many challenges ahead that need to be resolved for the full transition of this CRISPR/Cas technology from a preclinical study to a practical antiviral therapy, complete curative potential of the CRISPR/Cas-based antiviral strategy should provide continuous motivation for the development of novel antiviral therapeutics. Without a doubt, this new antiviral approach should help chronically infected patients stop taking a life-long medication in the near future.

## Figures and Tables

**Table 1:** Summary of CRISPR/Cas9-based antiviral studies targeting HIV-1. These anti-HIV studies were sub-classified based on their antiviral mechanism of actions. Abbreviations used within the table are as follows. CRISPR/Cas; clustered regulatory interspaced short palindromic repeat-associated nucleases, saCas9; Cas9 from *Staphylococcus aureus*, Cas9n; Cas9 nickase, gRNA; guide RNA, LTR; long terminal repeat, HIV-1; human immunodeficiency virus-1, GFP; green fluorescence protein, HEK293T; human embryonic kidney 293 cells with SV40 large T antigen, TNF-α; tumor necrosis factor alpha, 5-Aza-dC; 5-aza-2′-deoxycytidine, TSA; trichostatin A, AAV; adeno-associated virus, LV; lentivirus, PBMC; peripheral blood mononuclear cell, PMA; phorbol 12-myristate 13-acetate, Tat; tetracycline, Luc; luciferase, NOD/SCID; non obese diabetic-severe combined immunodeficiency, SAHA; suberoylanilide hydroxamic acid, HSF1; heat shock factor 1, SAM; synergistic activation mediator, CCR5; chemokine receptor type 5, HDAC; histone deacetylase, NF-kB, nuclear factor kappa B, CCR2; chemokine receptor type 2, iPSC; induced-pluripotent stem cell, CXCR4; C-X-C chemokine receptor type 4, IFN; interferon, ISG; interferon-stimulated gene, PD-1; programmed death-1, CTLA-4; cytotoxic T-lymphocyte-associated protein 4, APOBEC3G; apolipo-protein B mRNA editing enzyme, catalytic polypeptide-like, Fluc; firefly luciferase, TRIM5 α; tripartite motif-containing protein 5 alpha, VSV; vesicular stomatitis virus, WT; wild type, RT; reverse transcriptase, TAR; trans-activation response, RRE; rev-response element, IRES; internal ribosomal entry site, N/A; not applicable, ↑; up-regulation, and ↓; down-regulation.

Mechanism of Action	CRISPR/Cas System	Delivery	gRNA Target	Virus Study Model	Cell or Animal	Effect on Virus	Effect on Host	Reference
Direct disruption of an HIV genome	CRISPR/Cas9	Transfection	LTR	Pseudotyped LTIG HIV-1 with LTR-driven GFP expression and latently integrated provirus	HEK293T, HeLa, and Jurkat c5 and c19 cells	GFP expression↓, latency reactivation by TNF-α or 5-Aza-dC/TSA↓, excision of provirus	No off-target cleavage	[3]
	CRISPR/Cas9	Transfection	LTR U3	Latently Integrated provirus with LTR-driven GFP and luciferase expression	CHME5 microglial, HeLa-derived TZM-bI, U-937 U1 monocyte, and J-Lat T cells	GFP expression↓, latency reactivation by TSA↓, viral load↓, p24↓, excision of provirus, immunization against new infection	No effects on cell viability, no off-target cleavage	[4]
	CRISPR/Cas9	Transfection	Gag, env, pol, vif, rev, LTR	Lentivirus with a target gRNA sequence, latently Integrated provirus with LTR-driven GFP expression	HEK293T cells, primary human T cells, human pluripotent stem cell-derived macrophages and monocytes	Disruption of integrated lentivirus, GFP expression↓, p24↓, LTR-targeting gRNA LTR worked best, immunization against new infection	Cell viability↑, no off-target cleavage	[8]
	CRISPR/Cas9	Transfection	LTR, pol, and tet/rev	Latently integrated provirus with LTR-driven GFP expression	HIV-GFP Jurkat cell line called JLat10.6 cells	GFP expression↓, p24↓, tet/rev-targeting gRNA worked best	H3K9me2 histone modification↑	[12]
	CRISPR/saCas9	rAAV9 transduction	LTR and gag	HIV-1NL4–3 with a deletion of a 3.1 kb spanning the C-terminal of the Gag and the N-terminal of the Pol genes	HIV-1 Tg26 transgenic mice, rat, mouse embryonic fibroblasts, circulating rat lymphocytes	Viral RNA load in blood↓	N/A	[5]
	CRISPR/Cas9	Transfection and LV transduction	LTR U3	Latently Integrated provirus with LTR-driven GFP expression, HIV-1JRFL or PNL4-3	Human T-lymphoid cell line, 2D10, primary T cell, and patient-derived PBMC	GFP expression↓, latency reactivation by TSA and PMA↓, viral copy number↓, p24↓, excision of provirus, no reintegration, Immunization against new infection	No off-target cleavage, no effects on cell viability, cell cycle progression and apoptosis, and host gene expression	[6]
	Tat-inducible CRISPR/Cas9	Transfection and LV transduction	LTR	Latently Integrated provirus with LTR-driven GFP and luciferase expression	TZM-bl cells and human T-lymphocytic cells line, 2D10, Jurkat T-cells, human primary cultures of microglia and astrocytes	Expression of Tat by PMA and TSA, luciferase expression↓, GFP↓, viral load↓, excision of provirus, immunization against new infections	No cytotoxicity	[7]
	CRISPR/saCas9	LV transduction	LTR and gag/pol	EcoHIV-firefly luciferase reporter	HEK293 T cells	Luciferase expression↓, excision of provirus	N/A	[10]
	CRISPR/saCas9	LV and AAV transduction	LTR and gag/pol	EcoHIV-eLuc reporter	HEK293T cells, humanized bone marrow/liver/thymus (BLT) mice, HIV-1 Tg26 transgenic mice, neural stem and progenitor cells	Viral RNA load↓, Tat protein↓, luciferase expression↓, excision of provirus	No off-target cleavage, no AAV toxicity	[11]
	CRISPR/saCas9	LV transduction	LTR	HIV-1JR-FL	The in-vitro-infected PBMCs from HIV-1-positive patients embedded in the spleens of NRG and NOD/SCID mice and TZM-bl cells	Viral DNA and RNA load↓, p24↓	N/A	[2]
	CRISPR/saCas9	LV transduction	LTR and structural genes	HIV-1-expressing plasmid pNL4-3, latently Integrated provirus with LTR-driven GFP and luciferase	HEK293T, Jurkat C11, and TZM-bl cells	p24↓, GFP expression↓, luciferase expression↓, latency reactivation by SAHA↓, immunization against new infection	No off-target cleavage	[9]
Induction of latency reversal	dCas9-MS2-p65-HSF1-SAM	LV transduction	LTR	EcoHIV firefly-luciferase (eLuc) reporter, latently Integrated provirus with LTR-driven GFP	TZM-bI cell line, HEK293 T cells, HIV-1 latent T cell lines, CHME5 microglial cells, Jurkat-derived 2D10, and E4 cells	Luciferase expression↑, GFP expression↑, toxic viral proteins↑	Suicidal cell death in 2D10 and CHME5 cells	[30]
	dCas9-SunTag and dCad9-SAM	Transfection	LTR	HIV LTR-dependent luciferase reporter	HeLa cell-derived clonal TZM-bl cells, Jurkat-derived clonal JLat6.3 cells, Jurkat cell-derived HIV is cell line (HIVisB2), Jurkat-derived clonal J89 cells, MOLT-4/CCR5 cells	Luciferase expression↑, GFP expression↑, p24↑, infectious particles↑	N/A	[28]
	dCas9-SunTag-VP64	Transfection	LTR	LTR-luc, TZM-bl cells, a HeLa cell line integrated with a luciferase reporter expression cassette driven by HIV-1 5′-LTR, Jurkat T-cell-based latency models C11 cells (Latently Integrated provirus with LTR-driven GFP)	HEK293T cells, ACH2 cells, Jurkat T cells, C11, A10.6	GFP expression↑, luciferase↑, p24↑, binding of dCas9-SunTag-VP64 to LTR	No genotoxicity, global T cell activation, and cytotoxicity, no off-target cleavage	[31]
	dCas9-VP64, MS2-p64-HSF1-SAM, p300 (HDAC)	Transfection, LV transduction	LTR	HIV-1 subtype B promoter upstream of an EGFP reporter gene	HEK293T cells, Jurkat-derived lymphocytic cell lines J-Lat 9.2 and J-Lat 10.6 cells	GFP expression↑, synergy with SAHA and prostratin	N/A	[29]
	dCas9-MS2-p65-HSF1-SAM	Transfection	LTR	NL4-3.Luc.R-E-, a full-length HIV molecular clone where luciferase is driven by the viral LTR, LTRmCherry-IRES-Tat (LChIT) reporter	HEK293T cells, CEM T-cell, ACH2 cell, J-Lat cells	Luciferase expression↑, mCherry expression↑	No adverse effects, independent of NF-kB	[32]
Disruption of a host dependency factor	CRISPR/Cas9	Transfection	CCR5	N/A	K562 cells	N/A	N/A	[13]
	CRISPR/Cas9	Transfection	CCR5	N/A	HEK 293T cells	N/A	Deletion of CCR5 and CCR2 genes	[15]
	CRISPR/Cas9n	Transfection	CCR5	N/A	K562 cells	N/A	No off-target mutations	
	CRISPR/Cas9	LV transduction	CCR5	R5-trophic HIV-1	TZM.bl and CEMss-CCR5 cells, pseudo-type viruses with luciferase, human CD4 T CEMss-CCR5 cells	Resistant to R5-trophic HIV-1 infection, luciferase expression↓	CCR5 expression↓, no off-target mutations, selective survival advantage of CCR5-disrupted cells	[22]
	CRISPR/Cas9	Transfection	CCR5delta32	CCR5-tropic virus isolate, HIV-1SF170	iPSC differentiated into monocytes/macrophages	Resistant to R5-trophic HIV-1 infection	No off-target mutations	[24]
	CRISPR/Cas9	LV transduction	CXCR4	HIV-1NL4-3, a CXCR4-tropic HIV-1 with GFP driven by LTR	Ghost-CXCR4 cells, Jurkat cells and primary human and Rhesus macaque CD4+ T cells, Jurkat T cells	GFP expression↓, p24↓, viral RNA load↓	No genotoxicity or cytotoxicity, no off-target mutations	[16]
	CRISPR/Cas9	LV and adenovirus transduction	CCR5	HIV-1 BaL (R5-tropic) infection, Transmitted/founder (T/F) HIV-1 strains, Ad6F53 adenovirus vector	TZM-bl cells, primary CD4+ T-cells	Resistant to R5-trophic HIV-1 infection, luciferase expression↓, p24↓	No off-target mutations	[18]
	CRISPR/Cas9	Electroporation	CXCR4 and CCR5	CXCR4-tropic HIV-1LAI expressing GFP	Primary CD4 T cells	GFP expression↓	N/A	[17]
	CRISPR/Cas9	LV transduction and electroporation	CXCR4 and CCR5	HIV-1NL4-3 strain (X4-tropic) and HIV-1YU-2 strain (R5-tropic)	TZM-bl cell line, Jurkat T cells, primary CD4+ T cells	Resistant to R5-and X4 tropic HIV-1 infection, luciferase↓, p24↓	Selective advantage, no off-target mutations, no apoptosis difference	[20]
	CRISPR/saCas9	Transfection, LV and AAV transduction	CXCR4	LTR-GFP reporter, X4-tropic HIV-1NL4-3, HIV-1NL4-3	HEK293T cell and primary T cells, GHOST-X4 and TZM-bl cells, Jurkat T cells	Resistant to X4-trophic HIV-1 infection, GFP expression↓, p24↓, luciferase expression↓	No effects on cell viability, no apoptosis difference, no off-target mutations	[21]
	CRISPR/Cas9	Transfection	CCR5	Bal-1 virus (CCR5-tropic HIV-1 strain)	K562 cells, NOD/Prkdc-scid/IL-2Rγnull mice, CCR5-modified CD34+ hematopoietic stem/progenitor cells	Viral RNA load↓, resistant to R5-trophic HIV-1 infection	CCR5 ablation and reconstitution	[23]
	CRISPR/Cas9	Transfection	CXCR4-P191A	HIV LTR-dependent luciferase reporter, X4-tropic and R5-trophic HIV-1 strains	TZM-bl cells	Resistant to X4-trophic HIV-1 infection, viral RNA load↓, p24↓, luciferase expression↓	No effects on cell viability, No off-target mutations	[19]
	CRISPR/Cas9	LV transduction	miR-146	A latent infection cell model C11	HEK293T cells, A549 cells, MT2 cells, a latent infection cell model C11	Viral RNA load↓, p24↓, GFP expression by SAHA↓	No off-target mutations, NF-kB↑, NF-kB-regulated cytokines↑, Type I IFN↑, ISG↑, PD-1 and CTLA-4↓	[35]
	CRISPR/Cas9	LV transduction and electroporation	CXCR4 and CCR5	CXCR4-tropic HIV-1NL4-3 and CCR5 tropic HIV-1YU 2	GHOST (3) CXCR4+CCR5+ cells, primary human CD4+ cells, HeLa-CD4 cells	Resistant to X4-trophic and R5-trophic HIV-1 infection, p24↓	No effects on cell viability, no apoptosis difference, no off-target mutations	[25]
Induction of a host restriction factor	CRISPR/dCas9-SAM	Transfection	APOBEC3G and APOBEC3B	HIV-1 provirus containing the FLuc indicator gene in place of nef (HIV-1WTΔVif)	HeLa cells, 293T cells, CD4+ T-cell line CEMSS	Luciferase expression↓, infectivity↓	C2T mutation↑	[33]
	CRISPR/Cas9	Transfection	TRIM5αR332G and R355G	pHIV-1NL-GFP	293T cell, THP-1 cells, Jurkat cells	No HIV-1 restriction activity	Undesired mutations	[34]
Viral escape and resistance	CRISPR/Cas9	Transfection	LTR, *gag*, and *pol*	WT pNL4-3, NL-GFP, which is a VSV-G-pseudo-typed, GFP-expressing HIV-1, replication-competent WT HIV-1NL4-3	ACH-2 cells, MT-4 cells	GFP expression↓, p24↓↑	N/A	[36]
	CRISPR/Cas9	Transfection	LTR and entire protein-coding sequence	HIV plasmids pLAI	293T cells, SupT1 T cells	p24↓↑	Large virus-induced syncytia and cell death, mutations in the target for all escape viruses	[37]
	CRISPR/Cas9	Transfection and LV transduction	LTR and protein-coding sequence	HIV-1 LAI	293T cells	p24↓, no viral breakthrough	Large virus-induced syncytia and cell death	[38]
	CRISPR/Cas9	LV transduction	*Gag*/*pol*, env/rev, and LTR	NL4-3 HIV-1 strain, primary HIV-1 isolates 89.6 and YU-2, as well as three transmitted founder viruses CH040, CH077, and CH106	CD4+ SupT1 cells	Viral particles↓, delayed RT activity↑	N/A	[39]
	CRISPR/Cas9	LV transduction	TATA, TAR, RRE, env	HIV-1 strains NL4-3 and R7	Human CD4+ T cell line SupT1	p24↑	No effect on cell growth, cell viability↓	[40]
	CRISPR/Cas9	LV transduction	LTR, *gag*, and *pol*	LTR-GFP	J.Lat full-length clone 15.4, HIV-R7/E-/GFP	GFP expression↑↓, latency reactivation↓	N/A	[41]
	CRISPR/Cas9	Transfection	Tat, TAR, *gag*	NL-NLuc-HXB	293T cells, SupT1 cells	Luciferase expression↓, p24↓, resistance to a new infection	N/A	[42]

**Table 2:** Summary of CRISPR/Cas9-based antiviral studies targeting HBV. Abbreviations used within the table are as follows. HBV; hepatitis B virus, DHBV; duck hepatitis B virus, HBcAg; hepatitis B core antigen, HBsAg; hepatitis B surface antigen, NTCP; sodium-dependent uptake transporter, HBeAg; hepatitis B e antigen, cccDNA; covalently closed circular DNA, RFP; red fluorescence protein, rcDNA; relaxed circular DNA, NRG; non-obese diabetic-Rag(-)-γ chain(-), pgRNA; pregenomic RNA, HDI; hydrodynamic injection, IL-6, interleukin 6, pSTAT3; phosphorylated signal transducer and activator of transcription 3.

CRISPR/Cas System	CRISPR/Cas Delivery	gRNA Target	Virus AStudy Model	Cell or Animal	Effect on Virus	Effect on Host	Reference
CRISPR/Cas9	Transfection and HDI	PS, P1, XCp, eE, PCE, S1	HBV-expression vector pAAV/HBV1.2, DHBV-expressing plasmid, HBV-expression vector	Huh7 cells, C57BL/6 mice	HBcAg↓, HBsAg↓	N/A	[61]
CRISPR/Cas9	LV transduction	ENII-CP/X and Pre-C	HBV derived from the supernatant of HepAD38 cells	HepG2 cells expressing NTCP, HepAD38	HBcAg↓	N/A	[62]
CRISPR/Cas9	Transfection and HDI	X, C, P	pTHBV replication-competent plasmid, precccDNA, and pCre	Huh7 cells, HepG2.2.15 cells, BALB/c mice	HBsAg↓, HBeAg↓, cccDNA↓, HBcAg↓	N/A	[63]
CRISPR/Cas9nickase	Transfection	S and X	pRG-HBV double fluorescent reporter constructs	HeLa cells, HEK293 cells, stable HeLa and HEK293 cell lines containing integrated HBV-X or HBV-S reporter sequences, HepG2.2.15 and HepG2-H1.3., HepG2hNTCP	RFP↔, GFP↑, HBsAg↓, particle production↓	N/A	[64]
CRISPR/Cas9	LV transduction	RT, sAg, C	FLuc, integrated HBV genome	293 T-cells, HepAD38 and HepaRG, HBV2.2.15 cells	Luciferase expression↓, RT↓, HBV DNA↓, HBeAg↓, HBsAg↓, cccDNA↓, total DNA↓	N/A	[65]
CRISPR/Cas9	Transfection, LV transduction, and HDI	P-, S-, X-, and C	HBV genotype D replication-competent plasmid (pHBV1.3)	HepG2 cells, BALB/c mice	HBV mRNA↓, HBV DNA↓, rcDNA↓, replication intermediate↓, HBeAg↓, HBsAg↓, HBcAg↓	No cytotoxicity	[66]
CRISPR/Cas9	Transfection, LV transduction, and HDI	Core, pol, X, S	HBV-expressing plasmid	HepG2.2.15 cells, NRG mice	pgRNA↓, HBsAg↓, viremia↓, HBeAg↓, viral mRNA↓, core↓, cccDNA↓, de novo HBV infection↓	N/A	[67]
CRISPR/Cas9	Transfection	PreS/S, Enhl, X, preC/C	pBB4.5-HBV1.2, genotype C	HuH-7 cells, HepAD38 cells	HBsAg↓, HBeAg↓, HBV DNA↓, cccDNA↓	No cytotoxicity	[68]
CRISPR/Cas9	Transfection, LV transduction, and HDI	P-, S-, X-, and C	pAAV-HBV1.3	HepG2.2.15 cells, HDI in mice, HBV transgenic (HBV-Tg) model	HBsAg↓, cccDNA↓	N/A	[69]
CRISPR/Cas9nickase	Transfection	S, X, C	1.4XHBV DNA	HepG2, HEK293T cells	HBsAg↓, HBeAg↓, replicative intermediates↓, extracellular HBV DNA↓	No off-target mutations	[70]
CRISPR/Cas9	LV transduction	X and P	HBV from HepAD38 or 2.2.15 cells	HepG2/NTCP cells, HepAD38 cells	HBcAg↓	N/A	[71]
CRISPR/Cas9	Transfection	X and P	pcHBV1.3	Huh7 and HepG2 cells, M-TgHBV mice by HDI	HBsAg↓, HBeAg↓, HBcAg↓	N/A	[72]
CRISPR/Cas9	Lipid-like nanoparticles	S, X, C, P	1.3XHBV DNA	HepAD38 cells, mouse model by HDI	HBsAg↓, HBeAg↓, HBV DNA↓, HBV RNA↓	N/A	[73]
CRISPR/Cas9	Transfection	Repeated core region	Integrated HBV DNA	HepG2.A64	HBsAg↓, HBeAg↓, HBV DNA↓, cccDNA↓	No off-target mutations	[74]
CRISPR/saCas9	AAV	S and P	N/A	hNTCP-HepG2 cells, HepG2.2.15 cells	HBsAg↓, HBV DNA↓, pgRNA↓, viral particle↓, cccDNA↓	No off-target mutations	[75]
CRISPR/Cas9	Transfection and HDI	PreS, X, C, P	1.2×HBV and 3-2 binary	HepG2-NTCP-tet, HepAD38 cells, C57BL/6 mice	HBsAg↓, HBeAg↓, HBcAg↓, cccDNA↓	N/A	[76]
CRISPR/saCas9	AAV8 and HDI	S, P, C	pHBV-1.3B, prcccDNA/pCre, pAAV/HBV1.2	Huh7, HepG2.2.15 and HepAD38 cells, C3H mice	HBsAg↓, HBeAg↓, HBV DNA↓, pgRNA↓, cccDNA↓, rcccDNA↓	No off-target mutations	[77]
CRISPR/Cas9	High capacity AV	RT, P1, XCp	1.3 HBV genome containing plasmid pTHBV2,28	HepG2.2.15 cells, HepG-NTCP, HEK293 cells	HBsAg↓, HBV DNA↓, cccDNA↓, HBV RNA↓	No off-target mutations	[78]
CRISPR/Cas9nickase	Transfection	PreS1, S2, S	N/A	HepG2-2.15, PLC/PRF/5, Hep3B, xenograft mouse	HBsAg↓	Proliferation↓, tumorigenicity↓, IL-6↓, pSTAT3↓	[79]

**Table 3:** Summary of CRISPR/Cas9-based antiviral studies targeting herpes viruses including HSV-1, EBV, and HCMV. Abbreviations used within the table are as follows. HSV-1; herpes simplex virus-1, PML; promonocytic leukemia, LAT; latency-associated transcript, EBV; Epstein Barr virus, EBNA; EBV nuclear antigen, LMP; latent membrane protein, DsRed; red fluorescent protein, HCMV; human cytomegalovirus, IE; immediate early, OriP; origin of replication.

Target Virus	CRISPR/Cas System	CRISPR/Cas Delivery	gRNA Target	Virus Study Model	Cell or Animal	Effect on Virus	Effect on Host	Reference
HSV-1	CRISPR/Cas9	Transfection and LV transduction	ICP0, ICP4, ICP27	HSV-1	Vero cells, ICP0-complementing L7 cell line27, TC620 cells	Virus titer↓, HSV-1 infection↓, protein expression↓, HSV-1 replication↓, resistant to new infection	PML restoration, no off-target mutations	[89]
	CRISPR/Cas9	Transfection	UL7	HSV-1 strains 8 F	Vero cells, 293 T cells, BALB/c mice	Replication↓, in vivo virulence↓, viral load↓, LAT mRNA↓, IE a-4 transcription↓	Inflammation↓	[88]
EBV	CRISPR/Cas9	Nucleofection	EBNA, LMP	N/A	Burkitt’s lymphoma cell lines Raji, Namalwa, and DG-75	Viral load↓	Cell proliferation↓, apoptosis↑	[91]
	CRISPR/Cas9	Transfection	Bart promoter	GFP-expressing BX1 strain of EBV	HEK293-BX1 cells, AGS1-BX1, C666-1, and NP460-EBV	miR-Bart3↓, viral yields↓, insertion of DsRed	N/A	[92]
	CRISPR/Cas9	Transfection	EBNA1, OriP and W repeats	N/A	Nasopharyngeal carcinoma C666-1, HEK293M81 cells	EBV DNA↓, lytic replication↓, infection titer↓	Sensitized to chemotherapeutic killing	[93]
HCMV	CRISPR/Cas9	LV transduction	UL122/123 genes	TB40GFP and Toledo	MRC5 primary fibroblast cells, U-251 MG astrocytoma cells	IE protein expression↓, genome replication↓, late protein expression↓, particle production↓, envelope glycoprotein B↓	N/A	[95]
HSV-1, EBV, & HCMV	CRISPR/Cas9	LV transduction	miRNAs BARTs, EBNA1, EBV OriP	GFP-EBV, HCMV, HCMV: AD169	SNU-719, Burkitt’s lymphoma Akata-Bx1 cells, Vero cells, MRC5 cells	Escape variants, virus replication↓, viral breakthrough, viral titer↓, no effect on quiescence HSV-1 replication, reactivated HSV-1 from latency↓	N/A	[96]

**Table 4:** Summary of CRISPR/Cas9-based antiviral studies targeting HPV. Abbreviations used within the table are as follows. HPV: human papillomavirus. pRb: retinoblastoma tumor suppressor protein. PDX: patient-derived xenograft.

Target Virus	CRISPR/Cas System	CRISPR/Cas Delivery	gRNA Target	Virus Study Model	Cell or Animal	Effect on Virus	Effect on Host	Reference
HPV-16	CRISPR/Cas9	Transfection	E7	Split and reconstituted luciferase	SiHa, Caski, C33A, and HEK293 cells	E7 protein↓	Luciferase expression↑, apoptosis, and growth inhibition, pRb↑	[97]
HPV-18	CRISPR/Cas9	LV transduction	E6 and E7	Rev-gRNA target-GFP	293 T cells, HeLa cells, SiHa cells	N/A	GFP expression↓, p53↑, p21↑, pRb↑, Cell cycle arrest at G1, DNA replication↓, tumor cell death, no off-target mutations	[98]
HPV-16	CRISPR/Cas9	Transfection	E6/E7 promoter, E6, and E7	N/A	SiHa and C33-A, BALB/C nude mice	E6 mRNA↓, E7 mRNA↓	p53↑, p21↑, pRb↑, tumor growth↓	[99]
HPV-6 11	CRISPR/Cas9	Transfection	E7	N/A	Foreskin keratinocytes	E7 protein↓	Cell growth↓, apoptosis↑	[100]
HPV-16	CRISPR/Cas9	Transfection	E6 and E7	N/A	SiHa and C33-A	N/A	p53↑, p21↑, pRb↑, tumor growth↓	[101]
HPV-18	CRISPR/Cas9	Transfection	E6 and E7	N/A	SiHa and HeLa cells	N/A	p53↑, p21↑, pRb↑, cell growth↓	[102]
HPV-16	CRISPR/saCas9	AAV8	HPV-16 E6 and E7	N/A	293 T cells	N/A	PDX tumor volume↓	[103]

**Table 5:** Summary of CRISPR/Cas9-based antiviral studies targeting various viruses. Abbreviations used within the table are as follows. JCV: JC virus. T-Ag: T antigen. ASFV: African swine fever virus. PRV: pseudorabies virus.

Target Virus	CRISPR/Cas System	CRISPR/Cas Delivery	gRNA Target	Virus Study Model	Cell or Animal	Effect on Virus	Effect on Host	Reference
Polyomavirus JCV	CRISPR/Cas9	Transfection and LV transduction	T antigen	N/A	TC620 and SVG-A cells, BsB8 cells, HJC-2	T-Ag↓, viral replication↓, VP1↓, agnoprotein↓, colony number↓	No off-target mutations	[105]
ASFV	CRISPR/Cas9	Transfection	p30 (CP204L)	ASFV strain BA71V	WSL-gRp30 cells	Plaque formation↓, virus yield↓	No effects on cell growth	[107]
PRV	CRISPR/Cas9	Transfection	UL30	Pseudorabies virus (Suid herpesvirus 1, PRV)	PK-15 cells	PRV replication and yield↓↑, UL30↓, escape mutants	No off-target mutations	[109]
	CRISPR/Cas9	Transfection and LV transduction	75 sgRNAs	Luc Tag PRV	Vero cells, PK15 cells	Luciferase expression↓, virus titer↓↑,escape mutants	N/A	[110]
HCV	CRISPR/FnCas9	Tranfection	5’UTR, 3’UTR	HCVcc GT2	Huh7.5 cells	E2↓, luciferase↓, viral translation↓, replication↓	Independent on PAM	[113]
